# Old Yellow Enzyme from *Trypanosoma cruzi* Exhibits *In Vivo* Prostaglandin F_2_α Synthase Activity and Has a Key Role in Parasite Infection and Drug Susceptibility

**DOI:** 10.3389/fimmu.2018.00456

**Published:** 2018-03-07

**Authors:** Florencia Díaz-Viraqué, María Laura Chiribao, Andrea Trochine, Fabiola González-Herrera, Christian Castillo, Ana Liempi, Ulrike Kemmerling, Juan Diego Maya, Carlos Robello

**Affiliations:** ^1^Unidad de Biología Molecular, Institut Pasteur de Montevideo, Montevideo, Uruguay; ^2^Departamento de Bioquímica, Facultad de Medicina Universidad de la República, Montevideo, Uruguay; ^3^Programa de Farmacología Molecular y Clínica – ICBM, Facultad de Medicina Universidad de Chile, Santiago de Chile, Chile; ^4^Programa de Anatomía y Biología del Desarrollo – ICBM, Facultad de Medicina Universidad De Chile, Santiago de Chile, Chile

**Keywords:** *Trypanosoma cruzi*, prostaglandin F_2_α synthase, Old Yellow Enzyme, differentially expressed gene, benznidazol and nifurtimox activation

## Abstract

The discovery that trypanosomatids, unicellular organisms of the order Kinetoplastida, are capable of synthesizing prostaglandins raised questions about the role of these molecules during parasitic infections. Multiple studies indicate that prostaglandins could be related to the infection processes and pathogenesis in trypanosomatids. This work aimed to unveil the role of the prostaglandin F_2_α synthase *Tc*OYE in the establishment of *Trypanosoma cruzi* infection, the causative agent of Chagas disease. This chronic disease affects several million people in Latin America causing high morbidity and mortality. Here, we propose a prokaryotic evolutionary origin for *Tc*OYE, and then we used *in vitro* and *in vivo* experiments to show that *T. cruzi* prostaglandin F_2α_ synthase plays an important role in modulating the infection process. *Tc*OYE overexpressing parasites were less able to complete the infective cycle in cell culture infections and increased cardiac tissue parasitic load in infected mice. Additionally, parasites overexpressing the enzyme increased PGF_2_α synthesis from arachidonic acid. Finally, an increase in benznidazole and nifurtimox susceptibility in *Tc*OYE overexpressing parasites showed its participation in activating the currently anti-chagasic drugs, which added to its observed ability to confer resistance to hydrogen peroxide, highlights the relevance of this enzyme in multiple events including host–parasite interaction.

## Introduction

Chagas disease or American trypanosomiasis is an endemic zoonosis in South and Central America characterized by chronic inflammation and cardiomyopathy, and less frequently, digestive symptoms. Although disease pathogenesis remains unclear, it is well known that both parasite and host responses play relevant roles. Immediately after adhesion and during the initial stages of infection, *Trypanosoma cruzi* dramatically remodels host cell gene expression profile, with specific patterns on each cell type (1, 2); B and T cell immunity also plays important roles both in the control and pathogenesis of the disease (3, 4). The low or null parasite cardiac load in patients with chronic chagasic cardiomyopathy lead a discussion in the literature regarding the etiology of Chagas disease, where different factors such as *T. cruzi* strains, genetic background of the host, altered immune responses, and autoimmunity where associated with clinical outcomes of the disease [reviewed in Ref. (5)].

It was not until recently that bioactive lipids were recognized as relevant mediators of immune response to *T. cruzi* both during the acute (suppression of host lymphoproliferative responses to mitogens and antigens) and chronic (induction of inflammatory reactions in several tissues) phases of the disease (6–8). Prostaglandin F_2_α (PGF_2_α), thromboxane A_2_ (TXA_2_), prostaglandin I_2_ (PGI_2_), and prostaglandin E_2_ (PGE_2_) levels were shown to increase in infected mice (8, 9), and *T. cruzi*-derived eicosanoids were proposed as a mechanism of parasite persistence (10) as they are involved in disease evolution in favor of progression to the chronic stage (11, 12). In this sense, it was proposed that transition to the chronic phase is affected by the immunomodulatory effect of eicosanoids released by *T. cruzi*, which may contribute to parasite proliferation and differentiation, and also to host survival (11, 12). Furthermore, TXA_2_ and PGF_2_α were found to be the most abundant bioactive eicosanoids derived from *T. cruzi* during infection (11).

Although arachidonic acid (AA) metabolism in mammalian cells is well-described, prostaglandin pathways in trypanosomatids as well as the role of their derived eicosanoids in Chagas disease pathogenesis remain unclear. To date, the vast majority of characterized PGF_2_α synthases belong to the Aldo-Keto Reductase protein family (13). In trypanosomatids, *Tb*AKR (*Trypanosoma brucei*) and *Lm*AKR (*Leishmania major*) have PGF_2_α synthase activity (14, 15), whereas the *T. cruzi* ortholog *Tc*AKR seems to lack such activity (16). Interestingly, *T. cruzi* encodes a member of the Old Yellow Enzyme family (*Tc*OYE) that catalyzes PGF_2_α synthesis (11, 17). This flavoprotein NADPH oxidoreductase is absent in mammalian and even in other trypanosomatids (17, 18). Initially described by Warburg and Christian (19), Old Yellow Enzymes are oxidoreductases that use FMN as cofactor and can reduce nitro esters, nitroaromatics, or α,β-unsaturated compounds (20). Due to the diversity of enzymes belonging to OYE family and the wide variety of substrates identified, no conserved physiological role has been attributed to them. These enzymes have diverse functions associated to detoxification, oxidative stress response, and specific metabolic pathways such as ergot alkaloid biosynthesis (21).

*In vitro* experiments have shown that *Tc*OYE not only reduces 9,11-endoperoxide PGH_2_ to PGF_2_α and hydrogen peroxide but also is capable of metabolizing a number of trypanocidal drugs (17, 22). Although *Tc*OYE can reduce nifurtimox (Nfx) under anaerobic conditions, it is not able to reduce benznidazole (Bzn) (17). Nevertheless, deletion of *Tc*OYE gene copies as well as a decrease in *Tc*OYE transcription has been associated to Bzn resistance (23, 24).

The role of *Tc*OYE in host–parasite interactions has not been thoroughly assessed since most studies are focused on analyzing the *in vitro* activity of recombinant *Tc*OYE. Here, we studied the phylogenetic origin, expression patterns, subcellular localization, and overexpression effects of this enzyme evidencing that *Tc*OYE: (i) exhibits PGF_2_α synthase activity *in vivo*, (ii) modulates parasite progression into infective forms during the intracellular stages, (iii) augments parasitic load on mice heart muscle, and (iv) confers Bzn and Nfx susceptibility and hydrogen peroxide resistance.

## Results

### Phylogenetic Analysis of Old Yellow Enzyme Protein Family Members Reveals Presumptive Bacterial Origin of *Tc*OYE

**Figure 1:**
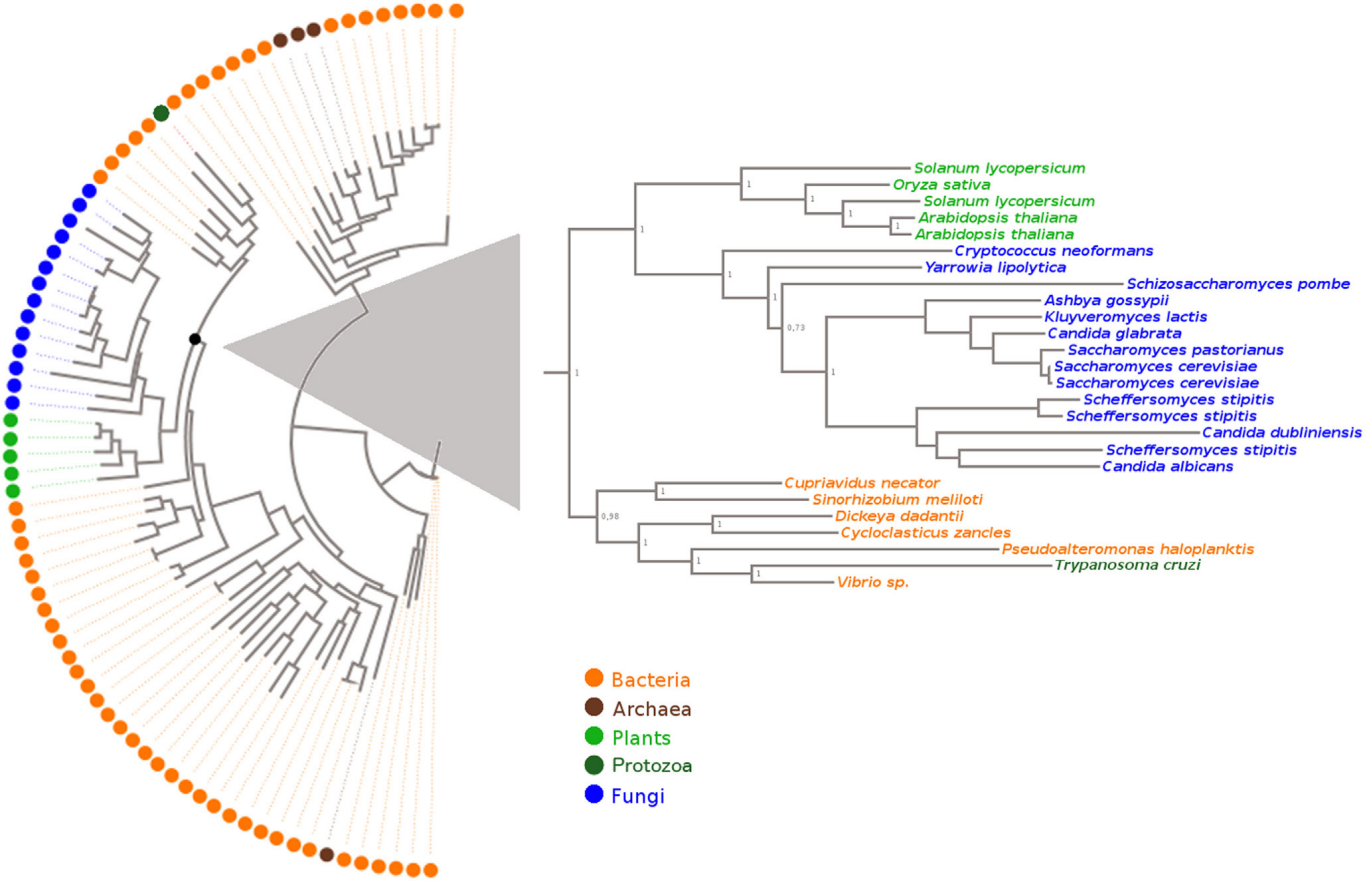
Evolutionary relationship of Old Yellow Enzyme proteins. The structure-based sequence alignment was performed using the accurate mode of T-Coffee software. The tree was built with the Maximum Likelihood method with WAG as best-fit model. Species name, protein ID, and KEGG/NCBI accession numbers of these sequences are shown in Table S1 in Supplementary Material.

To examine how *Tc*OYE is related to other proteins sharing sequence similarity, we performed a phylogenetic analysis (Figure 1) using proteins annotated as Old Yellow Enzymes in the KEGG Orthology database and manually recovered from published studies (multiple sequence alignment is shown in Figure S2 in Supplementary Material). NCBI-CD search (25) and Pfam (26) were used to confirm the presence of the characteristic protein domains (“TIM_phosphate_binding superfamily” and “Oxidored_FMN”) in the selected group of protein sequences. This analysis evidenced that OYEs clustered in two major lineages, one comprising proteins exclusively from Bacteria and Archaea, and another which included fungal, plant, protozoan, and also bacterial proteins. *Tc*OYE clustered in the group mainly formed by sequences from the phylum Proteobacteria, suggesting that *Tc*OYE possibly originated through horizontal gene transfer from this group of bacteria to *T. cruzi*.

### *TcOYE* Is Located in the Cytosol and Is Expressed in Epimastigotes and Amastigotes

**Figure 2:**
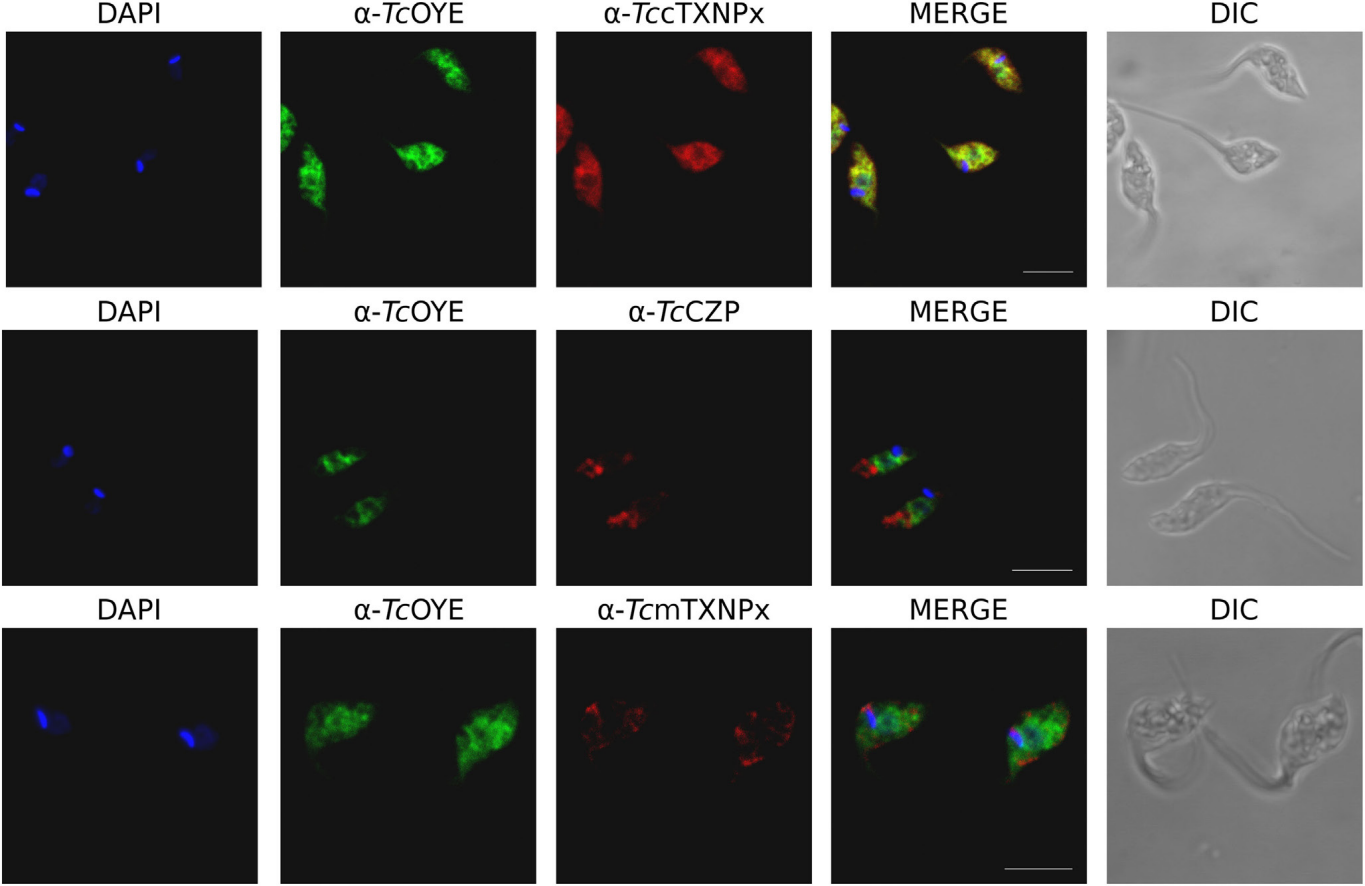
*Tc*OYE presents citosolic localization in epimastigotes. Immunolocalization of *Tc*OYE, *Tc*cTXNPx, *Tc*CZP, and *Tc*mTXNPx in epimastigotes using rabbit α-*Tc*OYE (1/3,000) and mouse α-*Tc*cTXNPx (1/100), α-*Tc*CZP (1/50), and α-*Tc*mTXNPx (1/100) polyclonal antibodies. Bar: 5 µm. DAPI was used as nucleus and kinetoplast marker.

*Tc*OYE subcellular localization was analyzed by indirect immunofluorescence (IIF) and western blot with differential membrane permeabilization assays. *Tc*OYE localization in epimastigotes is diffuse and cytoplasmic (Figure 2) in contrast to the granular appearance observed in early intracellular amastigotes (Figure S4 in Supplementary Material). In IIF assays *Tc*OYE presents co-localization exclusively with the cytosolic tryparedoxin peroxidase (*Tc*cTXNPx), but not with mitochondrial tryparedoxin peroxidase (*Tc*mTXNPx) and cruzipain (*Tc*CZP), mitochondrial, and reservosomal proteins, respectively (Figure 2). In addition, when epimastigotes were lysed with increasing concentrations of digitonin, *Tc*OYE displayed a similar pattern to *Tc*cTXNPx (cytosolic), and different from mitochondria (*Tc*mTXNPx), reservosome (*Tc*CZP), glycosome (*Tc*Glck, glucokinase), and endoplasmic reticulum (*Tc*APX, ascorbate peroxidase) markers, supporting a cytosolic distribution of *Tc*OYE in epimastigotes (Figure S5 in Supplementary Material).

**Figure 3:**
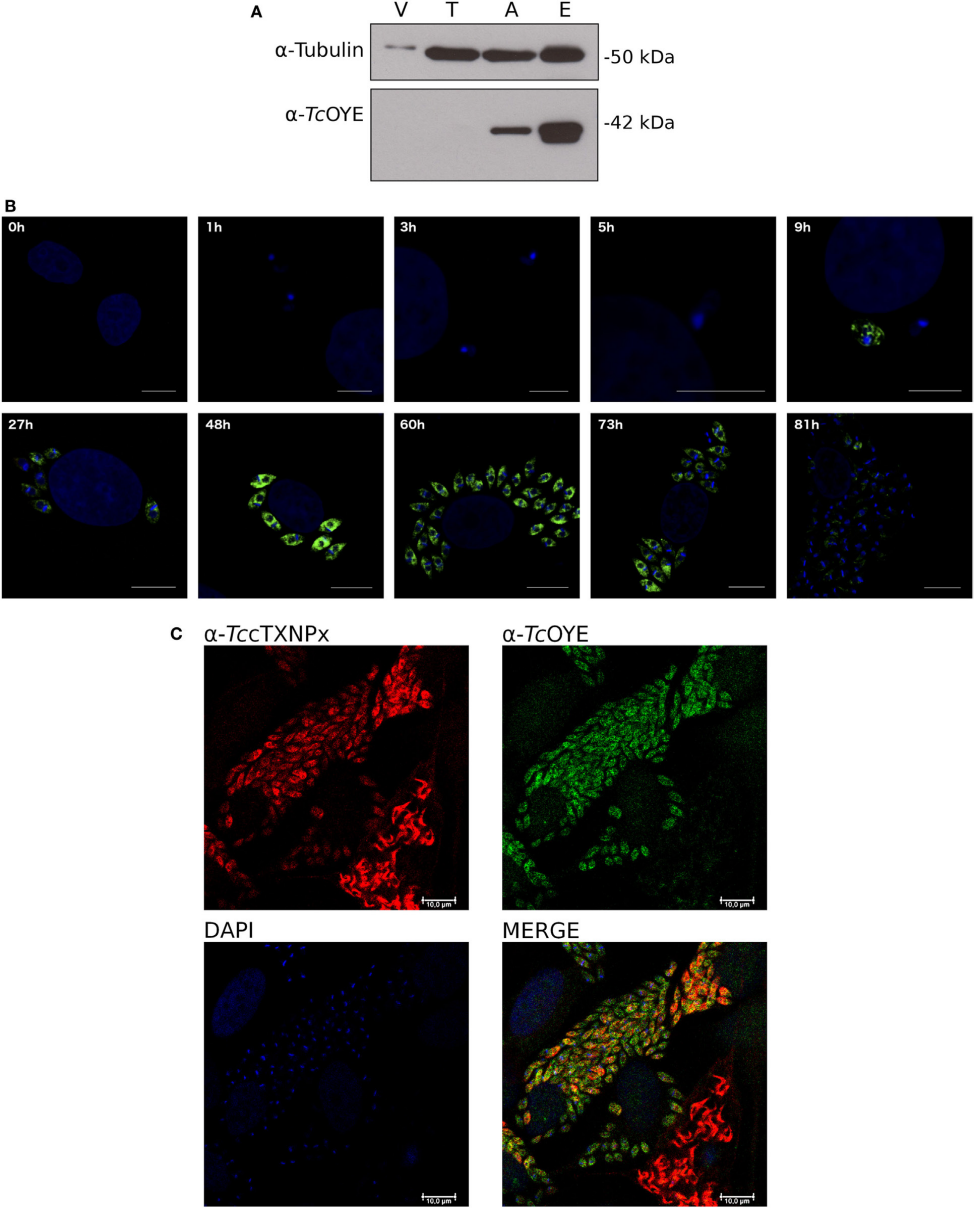
*Tc*OYE is expressed in replicative stages of *Trypanosoma cruzi* lifecycle. **(A)** *Tc*OYE expression during the parasite lifecycle was evaluated by western blot with total protein extracts from different parasite stages and Vero cells (since trypomastigotes and amastigotes are derived from infected Vero cells, they were used as a cross-reactivity control), using rabbit *Tc*OYE antiserum. Relative expression was estimated by densitometry normalized by tubulin expression. V, Vero cells, T, trypomastigotes, A, intracellular amastigotes, E, epimastigotes. **(B)** Kinetics of *Tc*OYE expression during mammalian cells infective cycle. Parasites were followed by inmunocitolocalization with rabbit α-*Tc*OYE polyclonal antibodies. Bar 10 µm (except 1–9 h 5 µm). **(C)** Confocal microphotographs showing *Tc*OYE and *Tc*cTXNPx immunolocalization in different stages of Vero cells infection. Bar 10 µm.

*Tc*OYE expression during *T. cruzi* lifecycle was analyzed by western blot using total extracts from different parasite stages (Figure 3A). A unique protein band of the expected size (42 kDa) was recognized by the polyclonal rabbit antiserum, confirming *Tc*OYE expression in epimastigotes and amastigotes, with 2.5-fold higher expression in epimastigotes. In contrast, the protein displayed undetectable levels in cell-derived trypomastigotes. The expression profile was further studied in the whole infective cycle by IIF (Figure 3B). *Tc*OYE expression was undetectable in early infection stages, and as parasites differentiated into amastigotes, *Tc*OYE expression increased gradually being maximal at 48 h post infection. Upon completion of the intracellular cycle, when parasites differentiate into trypomastigotes, the protein decreases again to undetectable levels. Furthermore, *Tc*OYE and *Tc*cTXNPx expression was compared in intracellular amastigotes and trypomastigotes mixed groups [being *Tc*cTXNPx a constitutive enzyme (27)]. Figure 3C shows that *Tc*OYE is expressed in amastigotes but becomes undetectable when these are differentiating into trypomastigotes, whereas *Tc*cTXNPx can be detected in both amastigotes and trypomastigotes.

### *Tc*OYE Overexpressing Parasites Increase PGF_2_α Production

**Figure 4:**
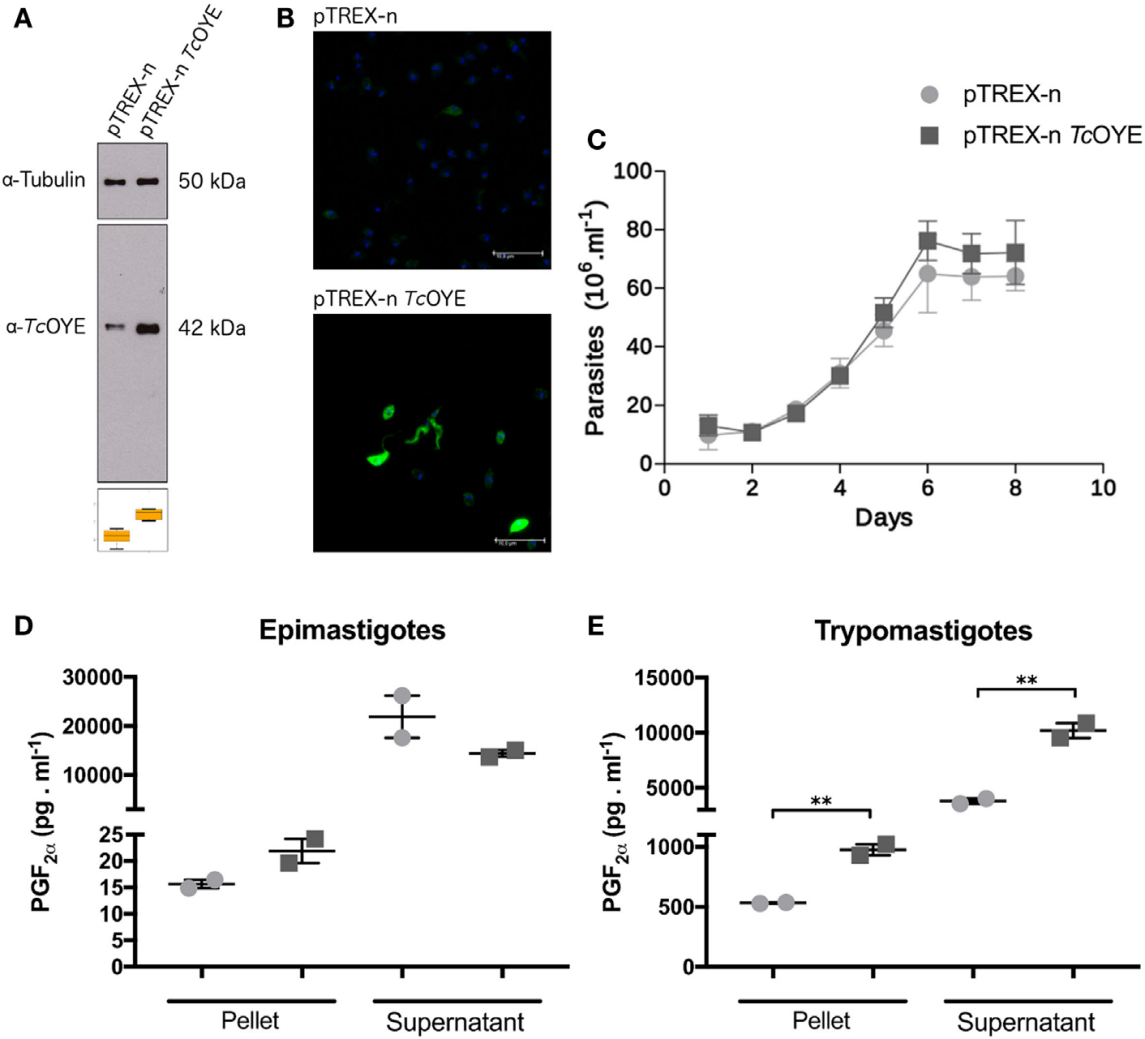
*Tc*OYE overexpressing parasites presents higher levels of PGF_2α_ than control parasites. **(A)** Western blot analysis of total protein extracts from epimastigotes transfected with pTREX-n (empty vector) and pTREX-n *Tc*OYE (includes complete *Tc*OYE coding sequence) using rabbit α-*Tc*OYE polyclonal antibodies. The relative expression was estimated by image densitometry analysis normalized by tubulin expression. *Tc*OYE overexpression in epimastigotes was 3.4 times more than controls. **(B)** Trypomastigotes and extracellular amastigotes overexpression analyzed by IIF using rabbit α-*Tc*OYE polyclonal antibodies. **(C)** Growth curves of *Tc*OYE overexpressing parasites (pTREX-n *Tc*OYE) vs. empty vector containing parasites (pTREX-n). **(D,E)** Determination of PGF_2α_ in epimastigotes and trypomastigotes. These results correspond to concentration values of PGF_2α_ from two replicates. Asterisks represent statistical significance (two tailed Student’s *t*-test).

To study the different aspects of *Tc*OYE involvement in *T. cruzi* infection process, overexpressing parasites were developed transfecting parasites with pTREX-n vector containing *Tc*OYE complete ORF. *Tc*OYE expression level was more than three times higher in overexpressing parasites than in control parasites (Figure 4A) and the overexpression was maintained along the entire cycle, including trypomastigotes (Figure 4B). Similar cell growth kinetics were observed for overexpressing parasites compared to controls, indicating that *Tc*OYE augmentation has no detrimental effect in epimastigotes proliferation (Figure 4C). To uncover if *Tc*OYE possesses PGF_2_α synthase activity *in vivo*, epimastigotes and trypomastigotes overexpressing *Tc*OYE were incubated with AA as a substrate for prostaglandin synthesis and PGF_2_α was measured in pellets and supernatants (Figures 4D,E). In both stages, *Tc*OYE overexpression correlates with an increase in PGF_2_α levels and this increase is statistically significant in overexpressing trypomastigotes compared to controls; possibly because basal activity in this life form is very low compared to epimastigotes. These results suggest that when the precursor is available *Tc*OYE acts as a PGF_2_α synthase in the parasite.

### *Tc*OYE Overexpression Confers Susceptibility to Bzn and Nfx and Resistance to Hydrogen Peroxide

**Figure 5:**
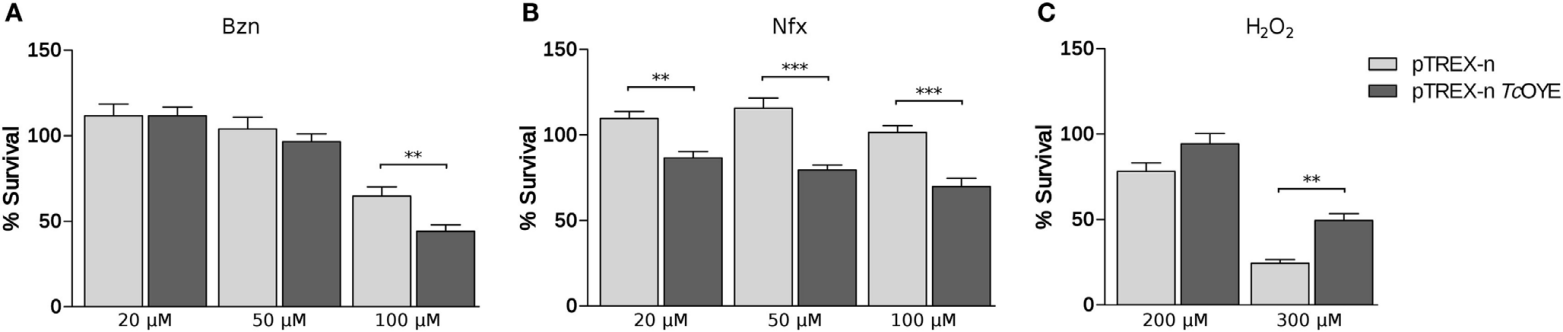
*Tc*OYE overexpressing parasites present differential viability to benznidazole (Bzn), nifurtimox (Nfx), or hydrogen peroxide (H_2_O_2_). **(A)** Viability percentages of transfected parasites challenged with different concentrations of Bzn. **(B)** Viability percentages of transfected parasites challenged with different concentrations of Nfx. **(C)** Viability percentages of transfected parasites challenged with different concentrations of hydrogen peroxide. The percentages of cell viability are normalized against parasites without treatment. Values are the means of three independent assays performed in quadruplicate. Asterisks represent statistical significance (two tailed Student’s *t*-test).

Since recombinant *Tc*OYE has been previously related to trypanocidal drugs metabolism (17, 24), we studied the effect of Bzn and Nfx treatment in *Tc*OYE overexpressing parasites. Transfected epimastigotes overexpressing *Tc*OYE were more susceptible to Bzn and viability decreased in all the assayed drug concentrations (Figure 5A). Besides, overexpressing parasites had higher sensitivity to Nfx than control parasites (Figure 5B).

Reduction of hydrogen peroxide (H_2_O_2_) by recombinant *Tc*OYE in anaerobic conditions has been previously described (17). To determine if *Tc*OYE participates in the redox metabolism, we exposed *Tc*OYE overexpressing epimastigotes to this oxidant. We observed a significant twofold increase in the viability of overexpressing parasites compared to controls (Figure 5C), evidencing that *Tc*OYE overexpression confers peroxide resistance. In addition, morphological changes evidenced in H_2_O_2_-treated control parasites were not observed in *Tc*OYE overexpressing parasites (Figure S6 in Supplementary Material). Furthermore, we observed H_2_O_2_ exposure increased *Tc*OYE expression in wild-type epimastigotes (Figure S7 in Supplementary Material).

### *Tc*OYE Overexpression Modifies *Trypanosoma cruzi* Infection Cycle *In Vitro*

**Figure 6:**
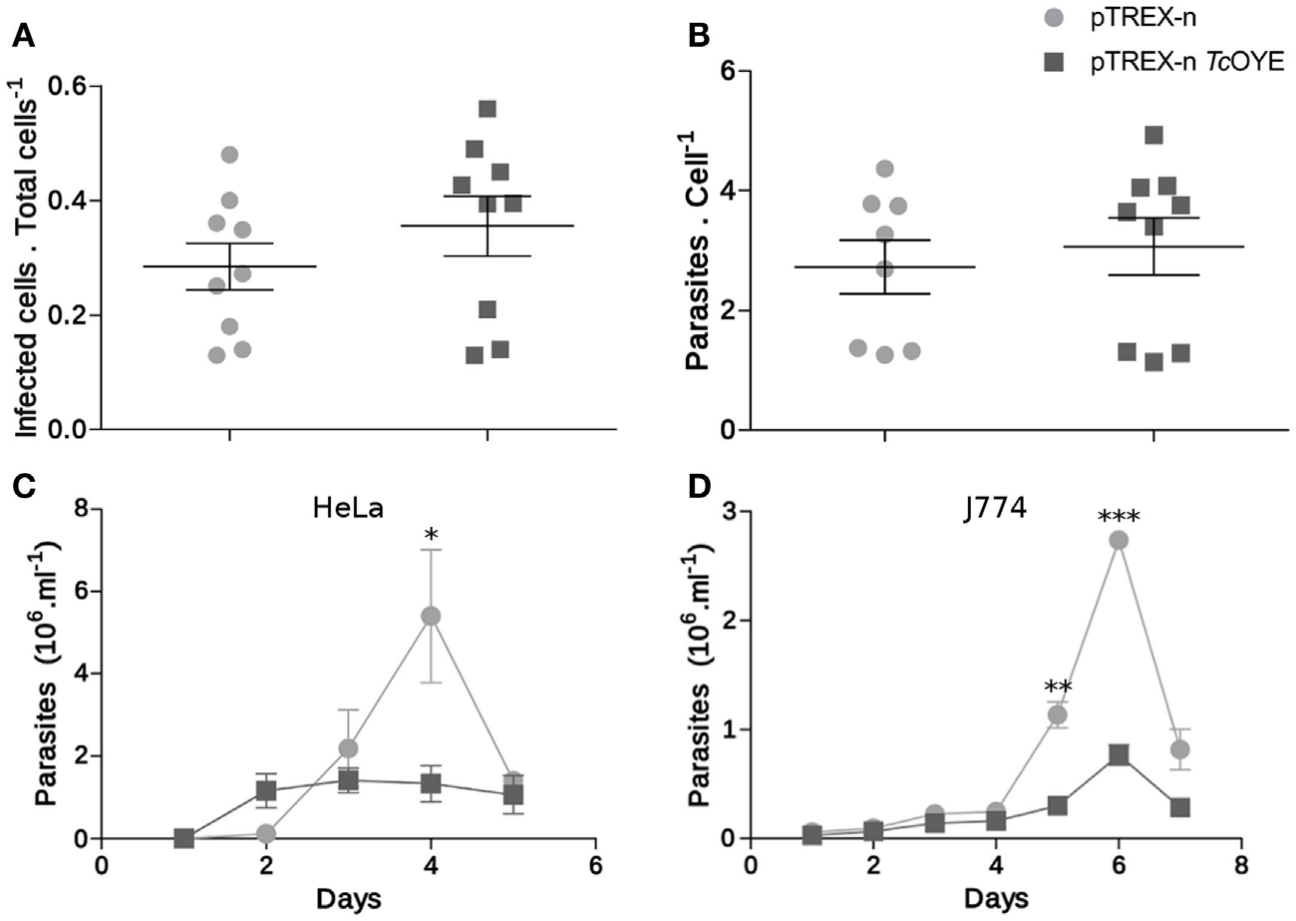
*Tc*OYE overexpression modulates parasite infective cycle. **(A)** *Trypanosoma cruzi* invasion capacity was not affected by *Tc*OYE overexpression. HeLa cells were incubated for 4 h with trypomastigotes and then the number of infected cells vs. total cells was evaluated. At least 200 cells per replicate were evaluated. No statistically significant differences were observed between the means of three independent assays performed in triplicates. **(B)** Intracellular amastigotes replication was not affected by *Tc*OYE overexpression. HeLa cells were incubated for 4 h with trypomastigotes and the number of internal parasites was counted 48 h post infection in at least 100 cells. No statistically significant differences were observed between the means of three independent assays performed in triplicates. **(C,D)** *Tc*OYE overexpression reduces the capacity of parasites to complete the infective cycle in different cell types. Number of trypomastigotes per milllilter of supernatant of HeLa cell or J774 macrophages infected with pTREX-n control parasites or pTREX-n *Tc*OYE transfected parasites. Asterisks represent statistical significance (two tailed Student’s *t*-test).

To get insight into the role of *Tc*OYE during mammalian cell infection, infectivity was evaluated *in vitro* in overexpressing and control parasites. There were no differences neither in the invasion ability nor in the replicative capacity of *Tc*OYE overexpressing parasites in comparison to controls (Figures 6A,B). Nevertheless, the amount of trypomastigotes released in the supernatant of infected cells was lower in transfectant parasites, suggesting that overexpression of the enzyme reduces the ability to complete the infective cycle. This effect was observed both in non-phagocytic and phagocytic cell lines (Figures 6C,D).

### Mice Infected with *Tc*OYE Overexpressing Parasites Showed an Early Parasitemia Peak and Higher Heart Muscle Parasitic Load

**Figure 7:**
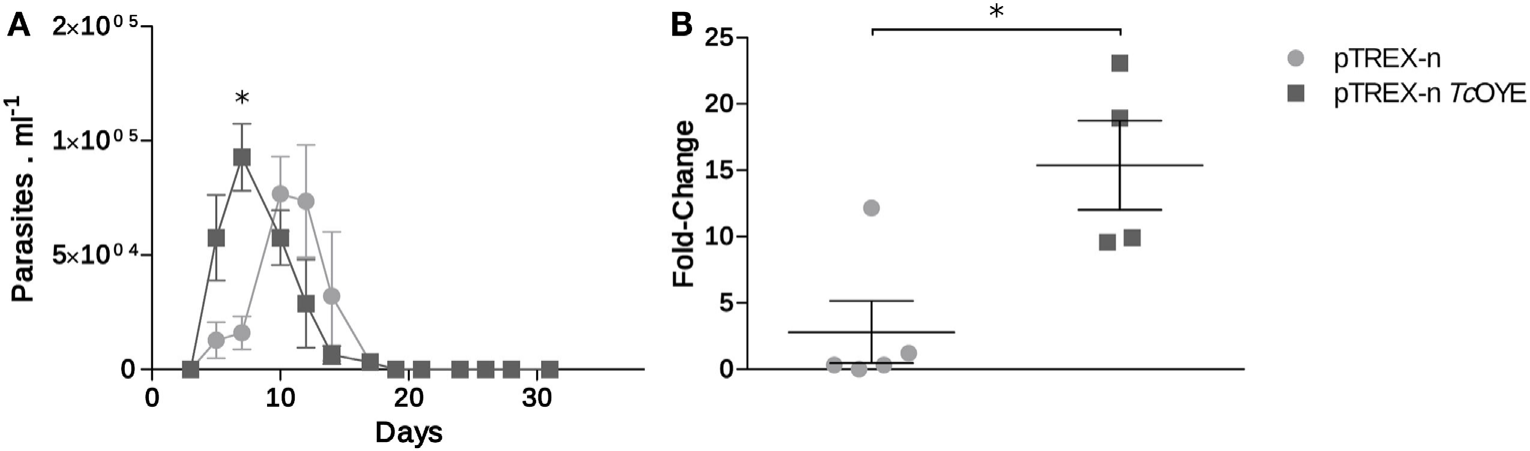
*Tc*OYE overexpression increased cardiac tissue parasite load in infected mice. **(A)** Parasitemia progression in BALB/c mice infected with *Tc*OYE overexpressing and control parasites. Parasitemia was measured every 3 days by microscopic examination of thin tail-blood smears. **(B)** Parasite load in cardiac tissue of mice infected with transfected *Trypanosoma cruzi* euthanized at the 30^th^ dpi. *N* = 2–5. Asterisks represent statistical significance (two tailed Student’s *t*-test).

Considering eicosanoids were described as immunomodulatory factors during parasitic infections (12), we studied the *Tc*OYE overexpression effect on parasitemia modulation and cardiac affection during mice infection. Control parasites produced a peak of parasitemia between 12 and 15 days post infection (dpi), while the peak of parasitemia in animals infected with *Tc*OYE overexpressing parasites occurred on day 6 (Figure 7A). Moreover, mice infected with *Tc*OYE transfectants presented an increased parasite load in the cardiac tissue (Figure 7B).

## Discussion

*Tc*OYE has been extensively studied, being most efforts focused on characterizing its reductive activity on nitroheterocyclic compounds (17, 22, 24, 28–32). Several roles have been attributed to *Tc*OYE, including drug metabolism, reduction of different compounds, and prostaglandin synthesis, although these reports were based on the non-infective insect-derived forms of the parasite or using the recombinant protein. However, little is known about its biological role and possible implication in host–pathogen interaction through *in vivo* models. In this work, we present a functional characterization of this enzyme which, together with related enzymes *Tc*AKR and *Tc*NTRI, are important proteins associated with the metabolism of the current anti-chagasic drugs (16, 17, 33). Our work is focused on poorly understood aspects of *Tc*OYE including its evolutionary origin, its role in mammalian infection, its expression and subcellular localization along the lifecycle, as well as its role as PGF_2_α synthase *in vivo*.

Considering that *Tc*OYE is only found in *T. cruzi*, and not in other trypanosomatids, we were interested in studying its evolutionary relatedness with representative eukaryotic and prokaryotic OYE proteins, to make inferences about its evolutionary origin and possible roles. To date, homologs have been identified only in bacteria (34), plants (35), fungi (19), and some protozoa (17, 36), having different roles depending on the organism. OYE proteins have been associated with oxidative stress response, detoxification of oxygenated lipids and with different specific metabolic pathways (21). Our phylogenetic characterization revealed that *Tc*OYE is phylogenetically closer to bacterial proteins than from other eukaryotes and, in particular, it formed a monophyletic group with Gammaproteobacteria indicating a possible gene transfer from this group of bacteria to *T. cruzi*. Among these organisms, *Dickeya dadantii* belongs to the family Enterobacteriaceae; and there are also several species of *Vibrio* which cause gastrointestinal tract diseases. Noteworthy, *T. cruzi* is stercoraria and during the epimastigote stage resides in a microenvironment with a high diversity of bacteria, a possible scenario for a horizontal gene transfer (37).

We studied *Tc*OYE expression along the *T. cruzi* life cycle using two different approaches demonstrating its highly regulated expression. *Tc*OYE was detected in replicative parasite forms (epimastigotes and amastigotes) but not in non-replicative cell-derived trypomastigotes. In particular, we found that *Tc*OYE is undetectable in trypomastigotes before mammalian cell infection and also when parasites start to differentiate into intracellular trypomastigotes, suggesting the protein is not relevant at the beginning and end of the intracellular infection cycle. Accordingly, *Tc*OYE (referred as dehydrogenase) has been identified in epimastigotes using alkaline bi-dimensional gels, whereas it was not detected when trypomastigote extracts were used (38). Also, the same differential protein pattern was observed by means of MudPIT proteomics (39). The lower or null *Tc*OYE expression in cell-derived trypomastigotes could be a consequence of mRNA downregulation at this stage, a differential protein translation or degradation. In this sense, using available data from two different transcriptomic analysis (2, 40), we compared the mRNA levels of *Tc*OYE (not shown) in different stages. Even though mRNA levels are higher in epimastigotes than in the other stages (in agreement with our observations at protein level), no significant differences were observed between amastigotes and trypomastigotes. These results suggest *Tc*OYE is being regulated by posttranscriptional mechanisms, which are widely used by these parasites. Regarding the role of *Tc*OYE in mammalian cell infection, later stages of the *in vitro* infection process were affected when parasites expressed *Tc*OYE constitutively (pTREX-n *Tc*OYE transfected line). Parasites were able to invade cells and replicate intracellularly but presented an impaired capacity to complete the infective cycle, since the amount of released trypomastigotes was reduced. This result, together with the observed reduction in *Tc*OYE levels during differentiation from amastigotes to trypomastigotes discussed above, suggested that a reduction in *Tc*OYE expression levels may be needed to fulfill the infective cycle. In a similar experiment using different *T. cruzi* strains, it was reported that *Tc*OYE overexpression causes a reduction in the number of intracellular amastigotes (32). Although in both cases the infection cycle is disturbed negatively, the differences could be attributed to different strains background. Recombinant *Tc*OYE is able to reduce PGH_2_ (11, 17) but, taking into account the wide range of substrates that can be reduced by this protein family on *in vitro* assays, we aimed to uncover if *Tc*OYE presents this activity in the parasite in presence of AA. In fact, PGF_2_α levels in *Tc*OYE overexpressing trypomastigotes were higher than in control parasites, demonstrating this enzyme is responsible for this activity in the parasite. Since *Tc*OYE expression is undetectable in trypomastigotes, we could determine significant differences in PGF_2_α synthesis among overexpressing and control parasites at this stage. This result also indicates that parasites can uptake the precursors from the extracellular media to produce its prostaglandins. Finally, a high level of PGF_2_α was found in culture supernatants, suggesting that this molecule is released from the parasite once it is synthesized, possibly to exert paracrine or endocrine effects.

Extensive studies on OYEs substrate specificity have suggested a role in detoxification and oxidative metabolism. The expression of OYE2 from *Saccharomyces cerevisiae* and YqjM from *Bacillus subtilis* is induced in response to oxidative stress and the exposure to toxic xenobiotics, evidencing an antioxidant role (34, 41). In *T. cruzi*, a peroxidase activity has been demonstrated *in vitro* for the recombinant *Tc*OYE (17). Accordingly, we observed an enhanced resistance to H_2_O_2_ in *Tc*OYE overexpressing parasites and an increased expression of this protein in wild-type parasites. In this context, *Tc*OYE could possibly acts as an antioxidant metabolizing lipid peroxidation products produced by H_2_O_2_ exposition, as previously described in *S. cerevisiae* (41). Although a pro-oxidant role has been proposed for this enzyme (32), our results showing an antioxidant role of *Tc*OYE are in agreement with the peroxidase activity described by Kubata et al. (17), and extensive evidence supports a protective role against oxidative stress conserved across the OYE family (34, 42, 43).

It has been proposed that recombinant *Tc*OYE uses NADPH to reduce Nfx under anaerobic conditions activating the prodrug (17). *Tc*OYE gene deletions were also associated with Bzn resistance induced *in vitro* (24) and recently *Tc*OYE overexpressing parasites were shown to have higher sensitivity to Bzn (32). Accordingly, we found that *Tc*OYE overexpression produced an increased susceptibility to both Bzn and Nfx. These results indicate that *Tc*OYE participates in the activation of Bzn and Nfx *in vivo*. In this sense, Wilkinson et al. demonstrated an increased resistance to Nfx and Bzn when both *Tc*NTRI and *Tb*NTRI, other type I nitroreductases, decreased their expression; whereas overexpression produced hypersensitivity (44). We demonstrated with functional studies that *Tc*OYE overexpression produced the same effect as *Tc*NTRI when the parasites are exposed to these prodrugs. Beyond its biological significance, *Tc*OYE is relevant given its nitroreductase activity and the ability to activate prodrugs that are used against Chagas disease.

*Tc*OYE overexpressing parasites used for infections in mice produced an earlier parasitemia peak and increased cardiac parasitic load. This result is relevant since myocardial damage due to persistence of the parasite is considered the most important mechanism in the development of chagasic cardiomyopathy (45). In addition, cardiomyopathy is considered one of the most important manifestations of *T. cruzi* infection (46). The reduced *Tc*OYE overexpressing parasites capacity to complete the lifecycle could explain the higher cardiac tissue parasitic load in infected mice. Nevertheless, more experiments are needed to understand the systemic mechanisms that are acting.

Several features uncover the importance of PGF_2_α activity for trypanosomatids. In particular, in *T. cruzi*, PGF_2_α is one of the most abundant prostanoid parasite-derived together with TXA_2_ (11). The reason why *Trypanosoma cruzi* has acquired an Old Yellow Enzyme with multiple functions and relevance in the host–parasite interactions as shown in this work, deserves further study.

## Materials and Methods

### DNA Amplification and Cloning

*Tc*OYE coding sequence was PCR-amplified from genomic DNA of *T. cruzi* Dm28c epimastigotes with Pfu DNA polymerase (*Fermentas*) usings EcoRI_Fw_OYE: (5′-AAGAATTCATGGCGACGTTCCCTGAACTTCTG-3′), HindIII_Rv_cSTOP_OYE: (5′-AAAAAGCTTTTAGTTGTTGTACGTCGGGTAATCGT-3′), and KpnI_FwOYE (5′-AAGGTACCATGGCGACGTTCCCTGAACTTC-3′) primers. PCR products were cloned in pGEM^®^-T Easy plasmid (*Promega*, USA) using T4 DNA Ligase (*Sigma*) and sequenced. EcoRI_Fw_OYE and HindIII_Rv_cSTOP_OYE primers were used to subclone into pTREX-n vector (47), and KpnI_FwOYE and HindIII_Rv_cSTOP_OYE for pQE30 vector (*Qiagen*).

### Expression and Purification of Recombinant Proteins

Recombinant 6His-tag fusion protein was expressed in M15 *Escherichia coli* strain. Cells were grown on LB medium supplemented with ampicillin (50 µg/ml) and kanamycin (25 µg/ml) at 37°C until OD600 nm ~0.6. Induction of protein expression was performed with 1 mM IPTG at 37°C for 4 h. Recombinant *Tc*OYE purification under native conditions was performed using FPLC A¨KTA™ purifier (*GE Healthcare Life Sciences*) in two steps: immobilized-metal affinity chromatography using HisTrap High Performance™ column (*GE Healthcare*) and anion exchange chromatography with a RESOURCE Q column (*GE Healthcare*). The recombinant proteins purity was analyzed by SDS-PAGE 12% stained with colloidal coomassie (Brilliant Blue G-250, *Sigma*), and protein concentration was determined by Bradford method (48).

Polyclonal antiserum against *Tc*OYE was obtained from New Zealand White rabbits after intraperitoneal injection of 100 µg of recombinant *Tc*OYE in Freund’s Complete Adjuvant (*Sigma*), followed by two immunizations with 50 µg of recombinant protein in Freund’s Incomplete Adjuvant (*Sigma*). Serum was obtained 15 days after the last boost.

### Western Blotting

Proteins resolved by electrophoresis in 12% acrylamide gels under reducing conditions were transferred to nitrocellulose membranes *Amersham™ Hybond^TM^-ECL* (*GE Healthcare*). Membranes were blocked for 2 h with blocking solution [3% (w/v) BSA, 0.1% (v/v) Tween 20 in PBS]; followed by 10 min in wash solution [0.1% (v/v) Tween 20 in PBS]. Membranes were incubated with antibodies diluted in 1% (w/v) BSA, 0.1% (v/v) Tween 20 in PBS for 3 h, washed three times with wash solution 10 min, and incubated for 1 h with HRP-conjugated goat anti-rabbit or anti-mouse secondary antibody (*Sigma* or *DAKO*, respectively). Finally, membranes were washed four times for 10 min with wash solution. The whole procedure was performed at room temperature. The signal was developed with Super Signal^®^ West Pico Chemiluminescent Substrate (*Thermo SCIENTIFIC, USA*) according to the manufacturer’s specifications. The normalization was performed with α-tubulin commercial antibody (*Sigma*, T5168) and bands were scanned and quantified using ImageJ 1.49m software. Rabbit *Tc*OYE antiserum was diluted 1/30,000.

### Parasites and Cells

Vero (49), macrophage-like cell line J774 (50), and HeLa (51) cells were cultivated in Dulbecco’s Modified Eagle’s Medium [DMEM(1×) + GlutaMAX™-l, *Gibco*^®^ *by Life Technologies*™] supplemented with 10% (v/v) fetal bovine serum (FBS, *Gibco*), penicillin 100 U/ml and 100 µg/ml streptomycin (Thermo SCIENTIFIC) at 37°C in a humidified 5% CO_2_ atmosphere.

*Trypanosoma cruzi* Dm28c (52) were cultured axenically in liver infusion tryptose medium supplemented with 10% (v/v) inactivated fetal bovine serum (*GIBCO*) at 28°C. Trypomastigotes were collected from the supernatant of infected monolayers of Vero cell lines and were maintained cyclically. Intracellular amastigotes were obtained from infected Vero monolayers using Iodixanol gradient. Briefly, infected cells were washed with cold PBS twice, resuspended in cold PBS supplemented with complete protease inhibitor cocktail, and scrapped. Cells were then lysed in a Dounce homogenizer. The homogenate (2 ml) was added slowly into 2 ml of iodixanol 16% in a 15-ml falcon and then centrifuged at 800 *g* for 15 min. The amastigotes enriched pellet was isolated and resuspended in cold PBS. The purity of the preparation was evaluated under a microscope.

*Trypanosoma cruzi* epimastigotes were transfected with pTREX-n (empty vector) or pTREX-n *Tc*OYE construction. Epimastigotes (8 × 10^7^) were resuspended in HBS Buffer (21 mM HEPES, 137 mM NaCl, 5 mM KCl, 6 mM glucose, pH 7.4) and electroporated with 100 µg of plasmid DNA using two pulses at 450 V, 1,300 μF, and 13 Ω in 4-mm cuvettes. Transfected parasites were then selected with increasing concentrations of G418 (*Sigma*) from 50 to 500 µg/ml. Overexpression was confirmed by western blot analysis and IIF using the Icy platform (http://icy.bioimageanalysis.org).

### DNA and Protein Extraction

DNAzol^®^ Reagent (*Invitrogen*) was used according to the manufacturer’s specifications for isolation of genomic DNA of *T. cruzi* Dm28c epimastigotes. The DNA was resuspended in sterile distilled water and stored at −20°C until use. Quantification was performed using a spectrophotometer NanoDrop™ 1000 (*Thermo SCIENTIFIC*).

To obtain total protein extracts from epimastigotes, amastigotes, and trypomastigotes from Dm28c strain, parasites were washed three times in cold PBS and one time with 10 mM Tris pH 7 to remove remnant salts. Then, 50 µl of lysis buffer [40 mM Tris base, 7 M urea, 2 M tiourea, 4% (w/v) CHAPS, 1 mM PMSF, 1% (w/v) DTT, 1× complete protease inhibitor cocktail (*Sigma*, REF 11873580001), nuclease mix] was added for each 1 × 10^6^ parasites. Lysates were vortexed for 1–3 min and then the solution was incubated 30 min at room temperature with gentle agitation to allow complete lysis. The lysates were centrifuged at 12,500 rpm for 30 min and the supernatant was stored at −80°C. Proteins were quantified with Bradford reagent (*Sigma*).

### Immunolocalization Studies

For IIF localization, parasites were fixed for 16 h at 4°C with 4% (w/v) paraformaldehyde and then incubated with 50 mM ammonium chloride (*Sigma*) for 10 min at room temperature. Parasite (1 × 10^6^) were settled in polylysine pre-treated slides and permeabilized 5 min with 0.5% (v/v) Triton-X100 (*Sigma*). Blocking was performed with 2% (w/v) BSA, 0.1% (v/v) Tween 20 in PBS for 1 h, and washing with 0.1% (v/v) Tween 20 in PBS. Cells were incubated with polyclonal antibodies anti-*Tc*OYE (1/3,000 dilution), anti-*Tc*cTXNPx (1/100), anti-*Tc*CZP (1/50), anti-*Tc*mTXNPx (1/100) primary antibodies for 1 h. After three washes, Alexa Fluor^®^ 488 goat anti-rabbit IgG (*Invitrogen*, A11034) and Alexa Fluor^®^ 546 goat anti-rabbit IgG (*Invitrogen*, A11010) or Cy3^®^ goat anti-mouse IgG (*Invitrogen*, M30010) secondary antibodies were added for 1 h at a 1/1,000 (v/v) dilution. After four washes, slides were mounted with Fluoroshield™ with DAPI (*Sigma*) and visualized under Leica TCSSP5 confocal microscope. The whole procedure was performed at room temperature.

For differential membrane permeabilization assays, 5 × 10^8^ epimastigotes were treated with increasing concentrations of digitonin (*AppliChem*) from 0 to 4 mg/ml (53). Parasites were washed once with PBS and twice with extraction buffer (20 mM Tris–HCl, 100 mM NaCl, 1 mM EDTA, 250 mM sacarose, pH 7.5). Parasites were resuspended in 850 µl of extraction buffer and 80 µl of this resuspension was incubated 5 min at 30°C with the corresponding digitonin dilution. Protein fractions were centrifuged for 10 min 15,500 *g* at 4°C, and supernatants were blended with loading buffer, boiled 5 min and conserved at −20°C. Protein extracts were employed in western blot analysis using different polyclonal sera as specific localization markers: *Tc*cTXNPx (cytosolic tryparedoxin peroxidase; cytoplasmatic; diluted 1/20,000), *Tc*Glck (Glucokinase; glycosomal; diluted 1/2,000), *Tc*mTXNPx (mitochondrial tryparedoxin peroxidase; mitochondrial matrix; diluted 1/2,000), *Tc*CZP (cruzipain; reservosome; diluted 1/1,000), and *Tc*APX (ascorbate peroxidase; endoplasmic reticulum; diluted 1/4,000).

### *T. cruzi* Mammalian Cell Infection and Invasion Assays

HeLa (30,000 cells/well) and macrophage-like cell line J774 (50,000 cells/well) cells were cultured onto 18-mm round glass coverslips in 12 wells plates (*Corning* Inc., Corning, NY, USA). Cells were infected with cell-derived trypomastigotes (pTREX-n or pTREX-n *Tc*OYE) at a ratio of five parasites per cell in DMEM without FBS. After 4 h of interaction, non-internalized parasites were removed by PBS washes and fresh DMEM supplemented with 2% (v/v) FBS was added. At different times coverslips were washed with PBS, fixed with 95% (v/v) ethanol and stained with Fluoroshield™ with DAPI (*Sigma*). Infectivity was assessed considering invasion, intracellular replicative capacity, and trypomastigote generation. Invasion capacity was evaluated by counting the number of infected cells after 4 h of interaction. Replication was analyzed as the number of amastigotes per infected cell at 48 h post infection. Internalized parasites were determined by Olympus IX81 microscope. Trypomastigotes completing the infective cycle, present in the supernatant every 24 h, were also counted.

### Susceptibility Experiments

*Tc*OYE overexpressing epimastigotes (5 × 10^6^) were washed twice with 1% (w/v) Glucose in PBS and then incubated 24 h with Bzn or Nfx (20, 50, and 100 µM) or 48 h with H_2_O_2_ (200 and 300 µM) in the same media. Parasites transfected with the empty vector were used as controls. The viability was evaluated with the resazurin reagent (R7017, *Sigma*) measuring absorbance at 490 and 595 nm. Results are referred to the condition of parasites without treatment.

### PGF_2_α Synthase Activity

*Tc*OYE overexpressing epimastigotes and trypomastigotes were washed with PBS and incubated with 50 µM of AA (ab120916, *Abcam*) in PBS for 2 h. Parasites were removed by pelleting at 1,100 *g* for 10 min. Parasite-free supernatant was stored at 4°C until PGF_2_α measurement with *PGF2 alpha High Sensitivity ELISA Kit* (ab133056, *Abcam*). The parasites were resuspended in PBS and an extract was performed by thermal shock (15 min at −80°C and 15 min at 37°C for three times consecutively). Finally, the resulting homogenized was centrifuged at 20,000 *g* for 30 min at 4°C and the supernatant was used for PGF_2_α determination. The measurements were performed in duplicate.

### Animal Infection

Intraperitoneal infection of 8-week-old male BALB/c mice was performed with *Tc*OYE overexpressing and control parasites. Mice were obtained from the Animal Facilities at the Faculty of Medicine, University of Chile and were maintained in a controlled environment under a 12-h day/night cycle at a constant temperature, with food and water available *ad libitum* as it was previously described by González-Herrera et al. (54).

Mice were intraperitoneally inoculated with 2 × 10^4^ blood trypomastigotes distributed in three groups: two mice were infected with wild-type parasites, five mice were infected with pTREX-n parasites and five mice were infected with pTREX-n *Tc*OYE parasites. Direct microscopic visualization of circulating trypomastigotes in thin tail-blood smears was used to evaluate *T. cruzi* infection every 3 days for a month. At 30th dpi, animals were euthanized and the hearts were obtained to evaluate parasite load in cardiac tissue. The hearts were conserved in 95% (v/v) ethanol for DNA extraction.

### Real-time PCR

Wizard Genomic DNA Purification Kit (*Promega*, USA) was used according to the manufacturer’s specifications for isolation of genomic DNA from heart samples from *T. cruzi*-infected BALB/c homogenized. qPCR reactions were performed in duplicate with SYBR^®^ Green PCR Master Mix (*Thermo Fisher Scientific*, USA) using TCZ1 (5′-CGAGCTCTTGCCCACACGGGTGCT-3′) and TCZ2 (5′-CCTCCAAGCAGCGGATAGTTCAGG-3′) primers, which amplify a 188-bp nDNA microsatellite region of *T. cruzi* genome (55). TNFα-5241 (5′-TCCCTCTCATCAGTTCTATGGCCCA-3′) and TNFα-5411 (5′-CAGCAAGCATCTATGCACTTAGACCCC-3′) primers were used as a loading control, since they are species-specific for *Mus musculus* a 170-bp sequence from TNFα gene. Real-time qPCR amplifications were performed using a 7300 Real-Time PCR system (*Applied Biosystems*, USA).

### Statistical Analysis

GraphPad Prism ^®^ Version 5.0 (GraphPad Software, Inc.) was used to determine statistically significant differences. Three technical replicates were assayed for each experiment, in at least two biological replicates. Data are present as mean ± SE. Statistical significance was assumed with probability values less than or equal to 0.05 using the following convention: *P ≤ 0.05; **P ≤ 0.01; ***P ≤ 0.001.

### Phylogenetic Analysis of Characterized Members of the Old Yellow Enzyme Protein Family

A representative set of well-known OYE (*n* = 78) sequences were obtained from KEGG Orthology database (56) and NCBI (Table S1 in Supplementary Material). Sequences with the characteristic domains of the family were used to perform phylogenetic and other comparative analyses. The protein sequences were multiply aligned using the accurate mode of T-coffee (57). This method takes into account the 3D structures available for improving the alignment quality. ProtTest v3.2.2 (58) was applied to find an optimal substitution model for each alignment. WAG (Whelan and Goldman) was the best-fit model. Phylogenetic analyses were performed with the maximum-likelihood method using the program PhyML (59). For the visualization FigTree v1.4.2 was used.

## Ethics Statement

The animal care and experimental infections were performed according to EU guidelines 1. All the experiments were performed by specialized researchers from the Faculty of Medicine at the University of Chile. All the protocols were revised and approved by the Committees on Bioethics from the Faculty of Medicine, University of Chile (Protocol CBA #0277 FMUCH).

## Author Contributions

Conceived and designed the experiments: FD-V, M-LC, AT, and CR. Performed the Molecular Biology experiments (cloning, expression of *Tc*OYE in *E. coli* and *T. cruzi*): FD-V, M-LC, and AT. Infection experiments on mice: UK, JM, FG-H, CC, AL, and FD-V. Data analysis: FD-V, M-LC, AT, FG-H, CC, AL, UK, JM, and CR. Wrote the manuscript: FD-V, AT, M-LC, and CR.

## Conflict of Interest Statement

The authors declare that the research was conducted in the absence of any commercial or financial relationships that could be construed as a potential conflict of interest.

## Supplementary Material

Table S1 Old Yellow Enzyme proteins.

Figure S1 Phylogenetic analysis of proteins annotated as Old Yellow Enzyme.

Figure S2 OYE sequences analysis. **(A)** Multiple sequence alignment of full length OYE proteins carrying a single domain from bacteria, archaea, plants, fungi, and protozoa using the accurate mode of T-coffee. The positions of the core active site residues are highlighted with the rectangular boxes. **(B,C)** Pfam and NCBI-CD search domain are highlighted in the full length OYE protein from *Trypanosoma cruzi* (*Tc*OYE).

Figure S3 Expression and purification of recombinant *Tc*OYE. SDS-PAGE 12% stained with colloidal coomassie. Recombinant 6His-tag fusion protein was expressed in M15 *Escherichia coli* strain. The protein purification was performed under native conditions in two steps: immobilized-metal affinity chromatography (IMAC) and anion exchange chromatography.

Figure S4 Immunofluorescence confocal images of intracellular amastigotes. Inmunofluorescence images of intracellular amastigotes using rabbit α-*Tc*OYE antiserum (1/3,000). Bar: 5 µm. DAPI was used as nucleus and kinetoplast marker.

Figure S5 Digitonin titration of *Tc*OYE in *Trypanosoma cruzi* epimastigotes. *T. cruzi* epimastigotes were permeabilized with increasing digitonin concentrations and the samples were evaluated by western blot using α-*Tc*OYE (1/30,000), α-*Tc*cTXNPx (1/20,000), α-*Tc*Glck (1/2,000), α-*Tc*mTXNPx (1/2,000), α-*Tc*CZP (1/1,000), and α-*Tc*APX (1/4,000) polyclonal antibodies.

Figure S6 Morphological changes observed during hydrogen peroxide treatment.

Figure S7 *Tc*OYE expression increase in parasites exposed to hydrogen peroxide. Western blot analysis of total protein extracts from wild-type trypomastigotes exposed to H_2_O_2_ using α-*Tc*OYE (1/20,000). T0, vehicle control (without H_2_O_2_); T1, 10 min; T2, 20 min; T3, 40 min.
