# Supplementary figures and images for: Old Yellow Enzyme from Trypanosoma cruzi Exhibits In Vivo Prostaglandin F2α Synthase Activity and Has a Key Role in Parasite Infection and Drug Susceptibility
Source: Front Immunol. 2018 Mar 7;9:456. doi: 10.3389/fimmu.2018.00456 (PMC5845897; doi:10.3389/fimmu.2018.00456)

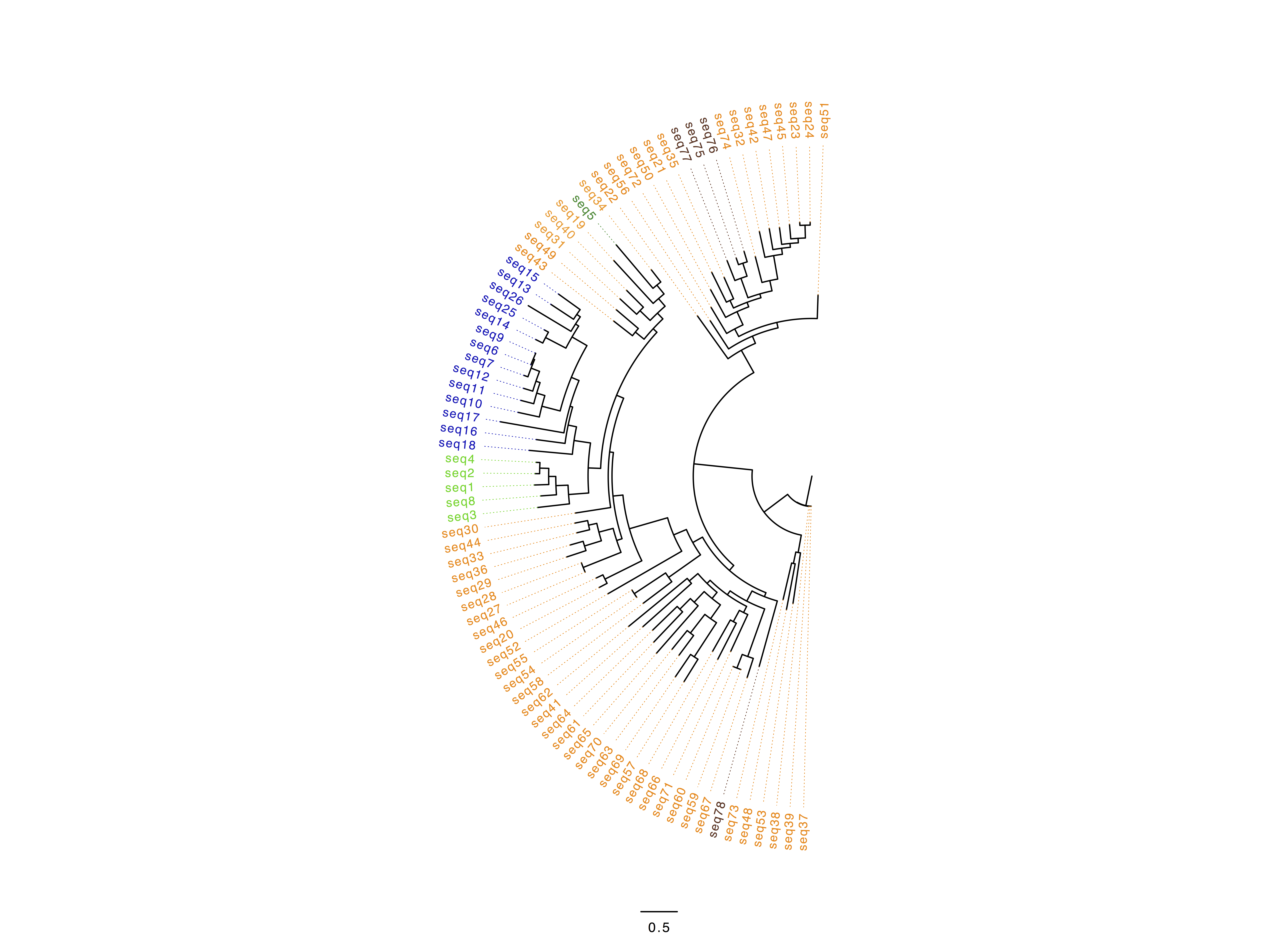

Supplement: Figure S1 — Phylogenetic analysis of proteins annotated as Old Yellow Enzyme. [file image_1.tiff]

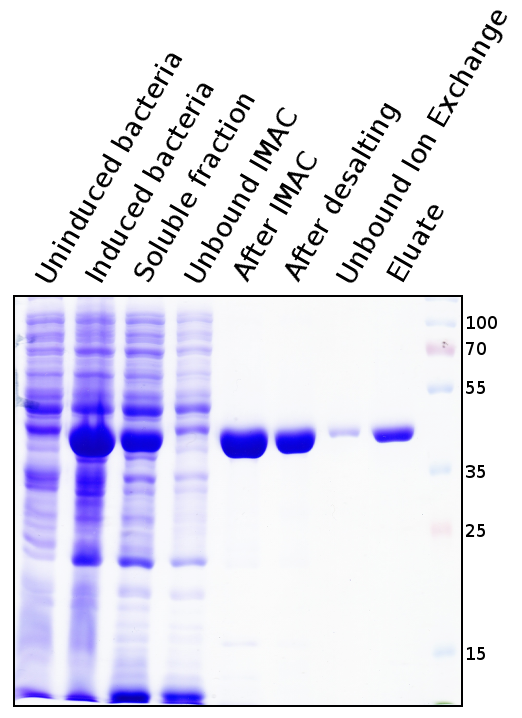

Supplement: Figure S3 — Expression and purification of recombinant TcOYE. SDS-PAGE 12% stained with colloidal coomassie. Recombinant 6His-tag fusion protein was expressed in M15 Escherichia coli strain. The protein purification was performed under native conditions in two steps: immobilized-metal affinity chromatography (IMAC) and anion exchange chromatography. [file image_3.tif]

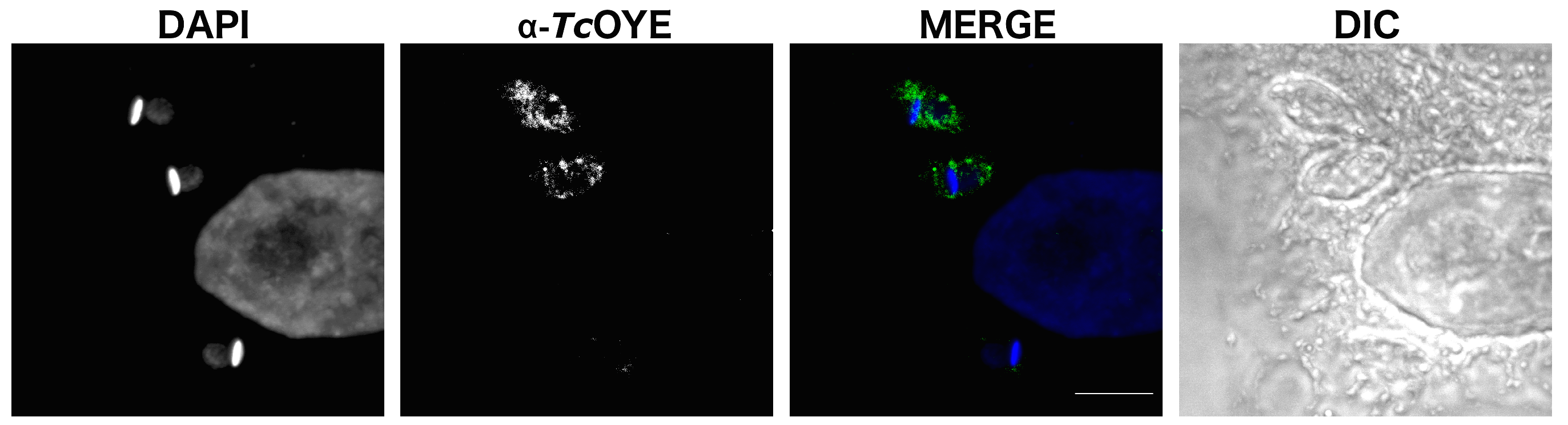

Supplement: Figure S4 — Immunofluorescence confocal images of intracellular amastigotes. Inmunofluorescence images of intracellular amastigotes using rabbit α-TcOYE antiserum (1/3,000). Bar: 5 µm. DAPI was used as nucleus and kinetoplast marker. [file image_4.tif]

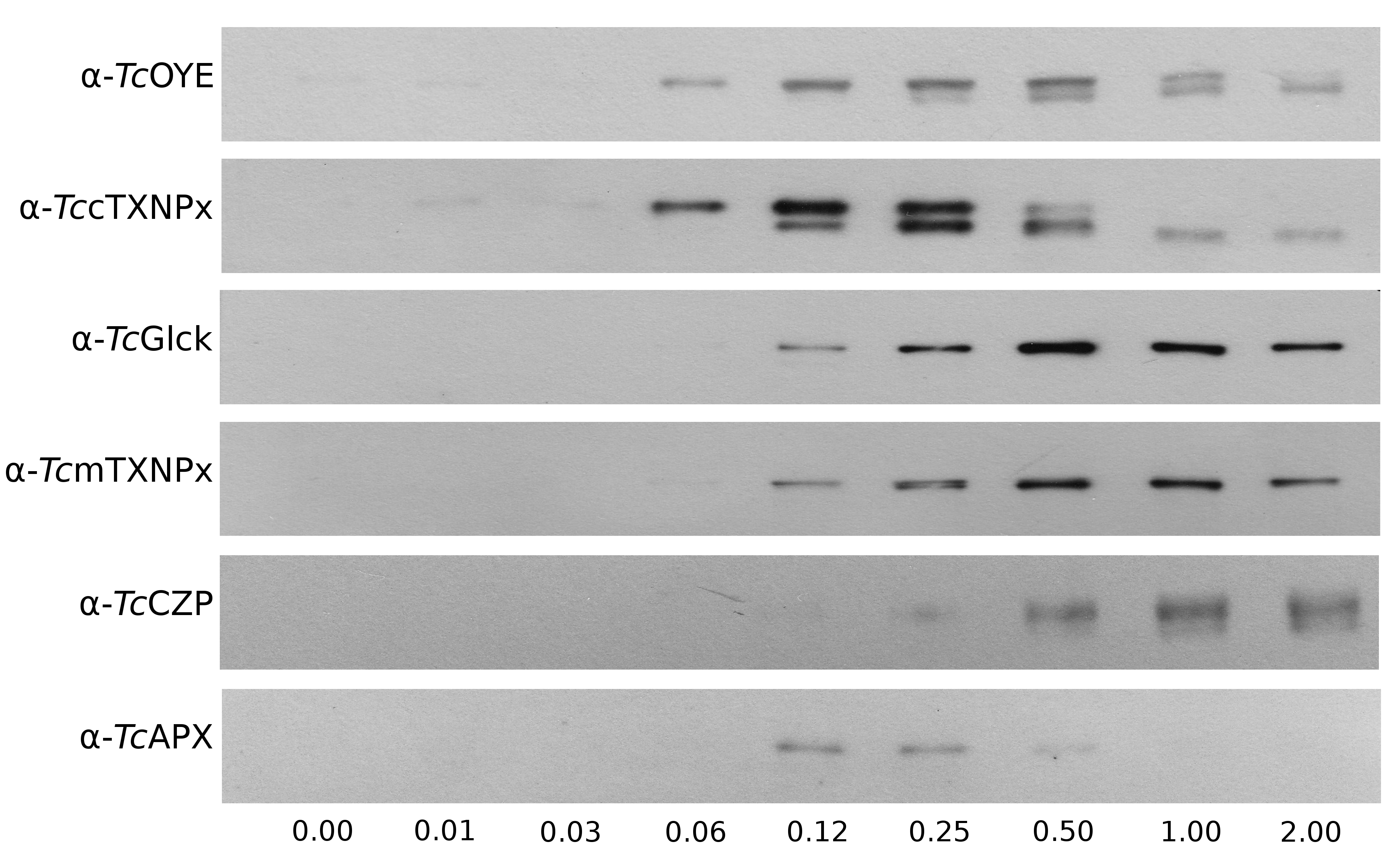

Supplement: Figure S5 — Digitonin titration of TcOYE in Trypanosoma cruzi epimastigotes. T. cruzi epimastigotes were permeabilized with increasing digitonin concentrations and the samples were evaluated by western blot using α-TcOYE (1/30,000), α-TccTXNPx (1/20,000), α-TcGlck (1/2,000), α-TcmTXNPx (1/2,000), α-TcCZP (1/1,000), and α-TcAPX (1/4,000) polyclonal antibodies. [file image_5.tif]

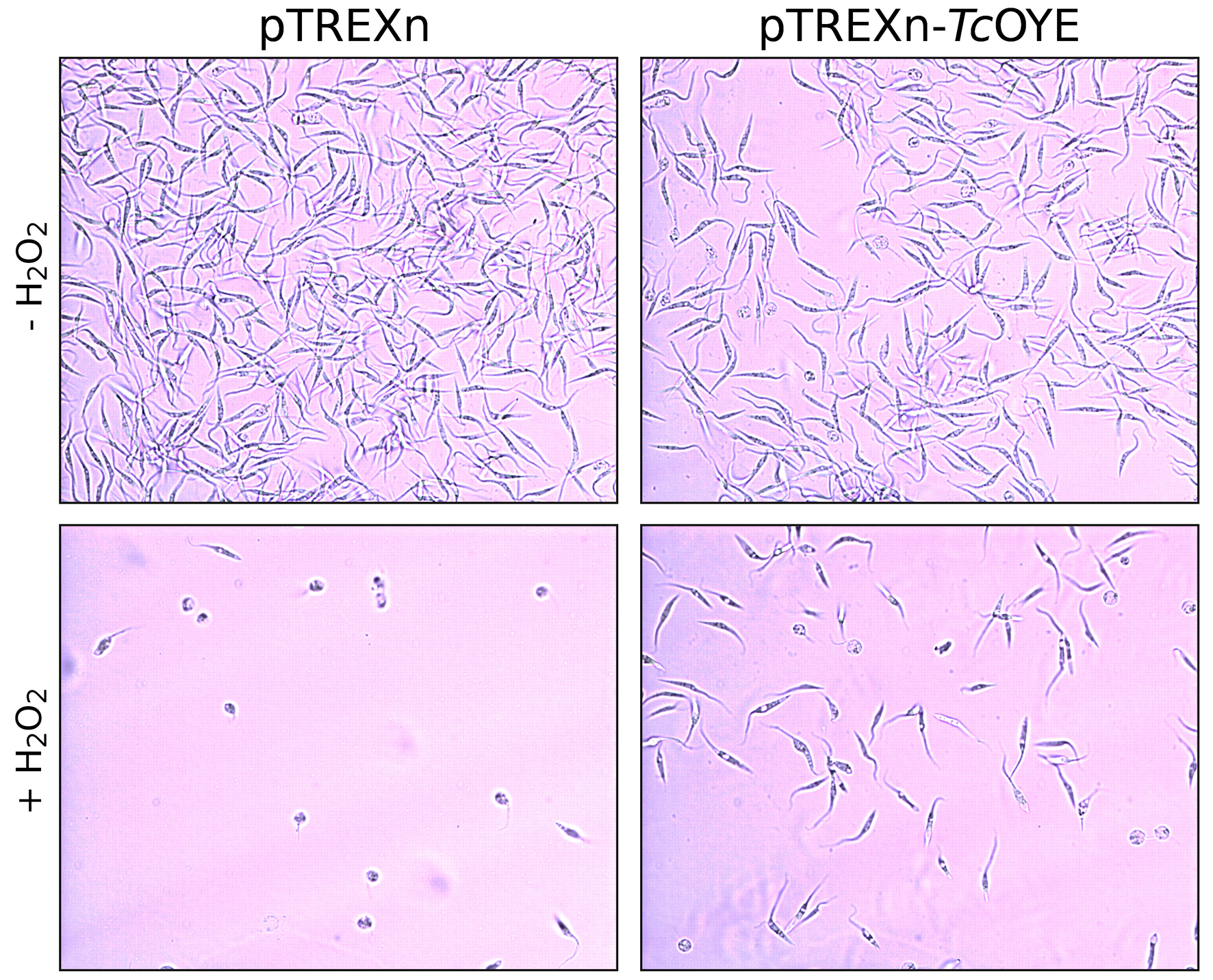

Supplement: Figure S6 — Morphological changes observed during hydrogen peroxide treatment. [file image_6.tif]

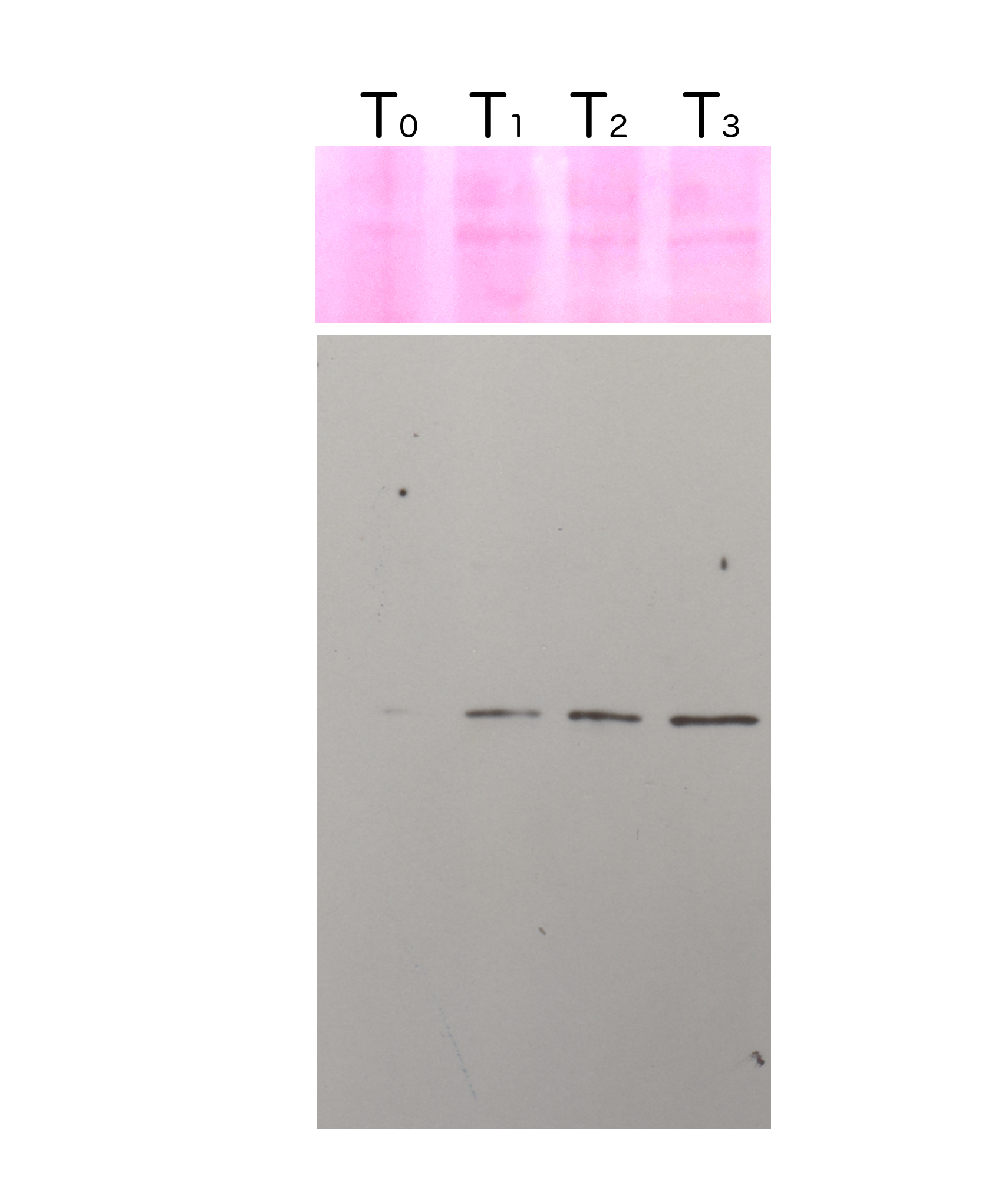

Supplement: Figure S7 — TcOYE expression increase in parasites exposed to hydrogen peroxide. Western blot analysis of total protein extracts from wild-type trypomastigotes exposed to H2O2 using α-TcOYE (1/20,000). T0, vehicle control (without H2O2); T1, 10 min; T2, 20 min; T3, 40 min. [file image_7.tif]
