# Supplementary material for: Old Yellow Enzyme from Trypanosoma cruzi Exhibits In Vivo Prostaglandin F2α Synthase Activity and Has a Key Role in Parasite Infection and Drug Susceptibility
Source: Front Immunol. 2018 Mar 7;9:456. doi: 10.3389/fimmu.2018.00456 (PMC5845897; doi:10.3389/fimmu.2018.00456)
Supplement: Figure S2 — OYE sequences analysis. (A) Multiple sequence alignment of full length OYE proteins carrying a single domain from bacteria, archaea, plants, fungi, and protozoa using the accurate mode of T-coffee. The positions of the core active site residues are highlighted with the rectangular boxes. (B,C) Pfam and NCBI-CD search domain are highlighted in the full length OYE protein from Trypanosoma cruzi (TcOYE). [file image_2.PDF]

A)

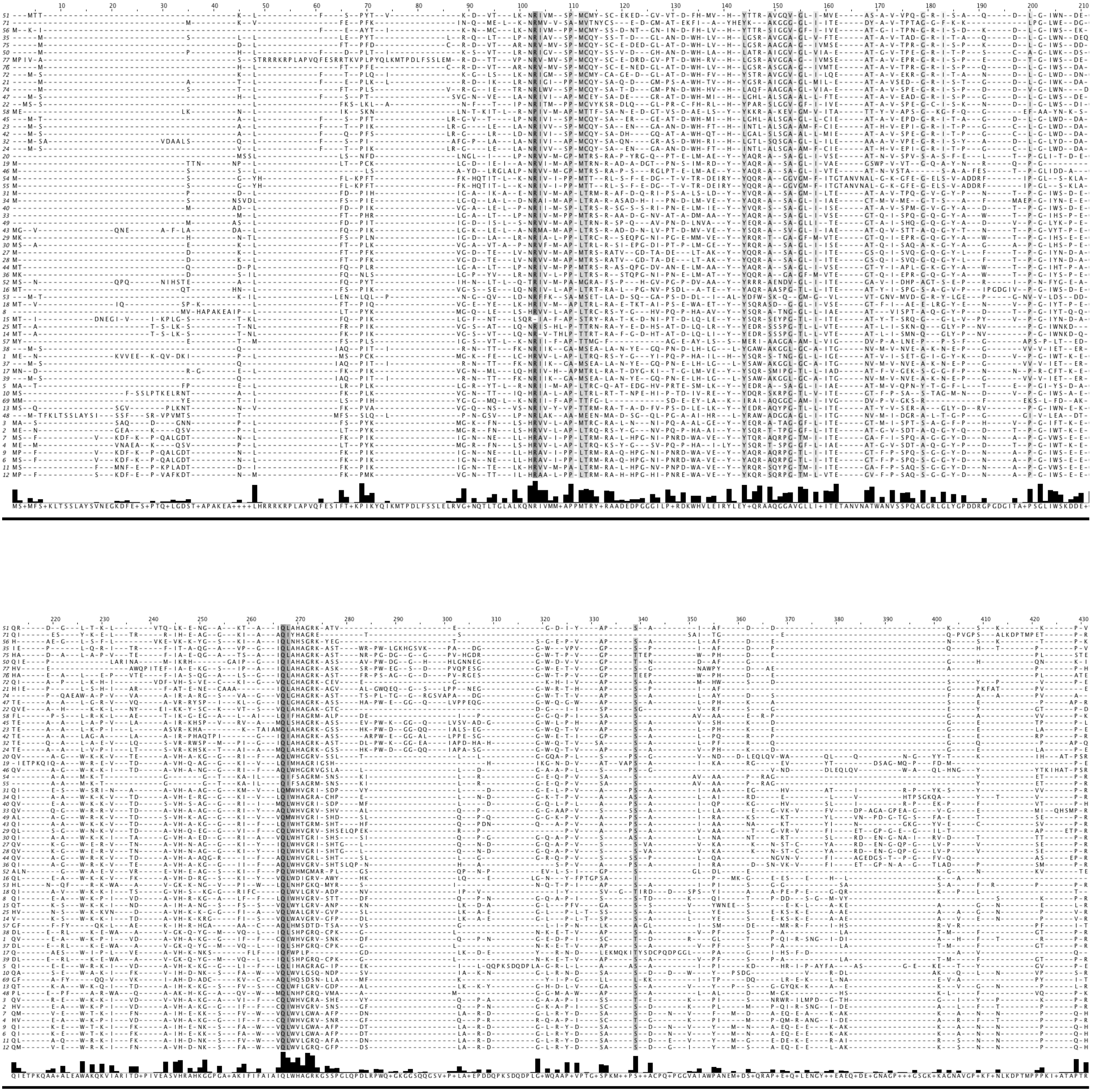

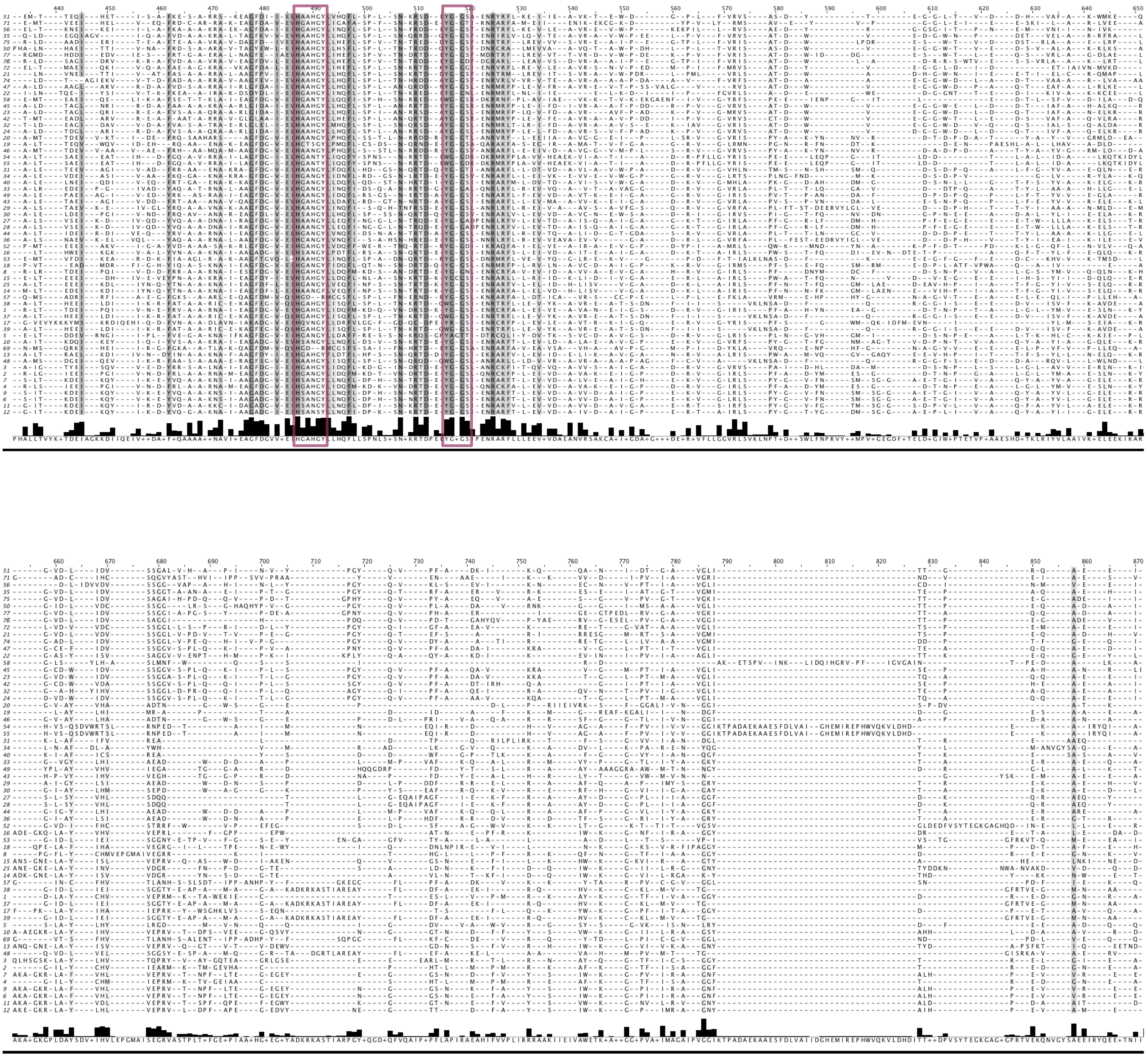

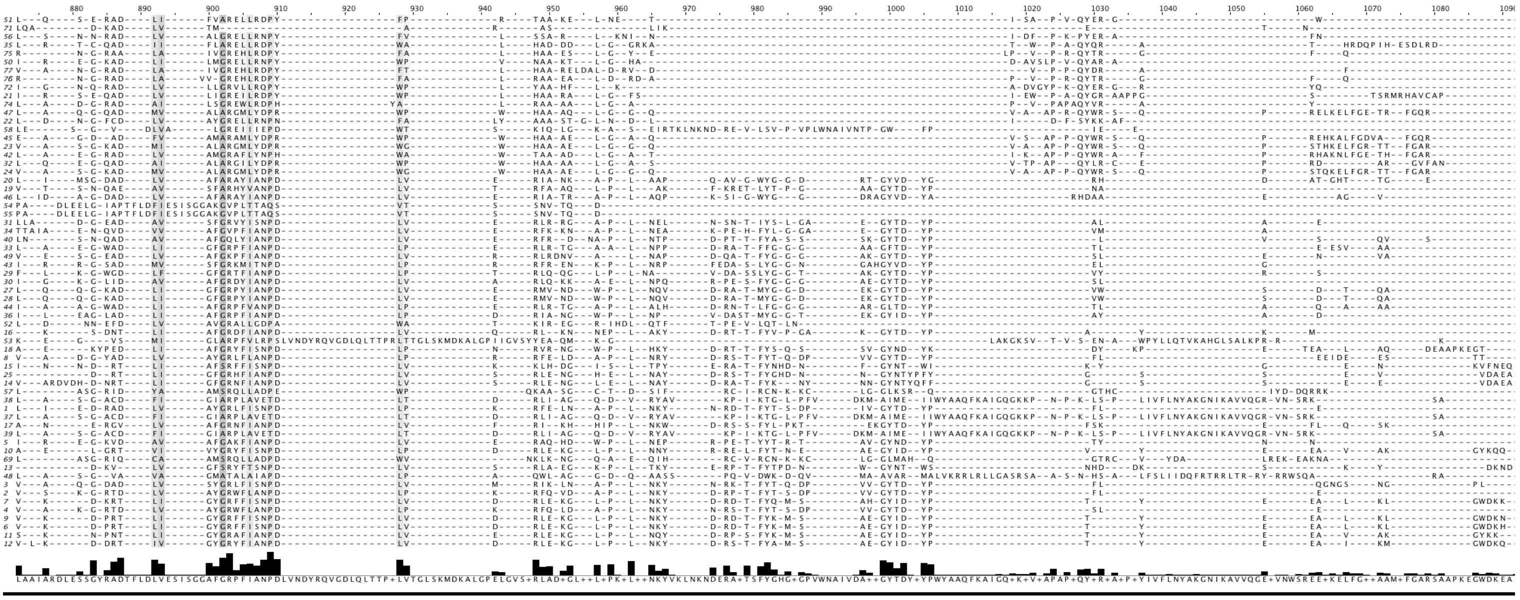

B) Pfam domain “Oxidored_FMN” from position 7 to 356, E-value 1,8e-67.

MATFPELLRPLKLGRYTLRNRIIMAPLTRCQATEDGHVPRTESMLKYYEDRASAGLIIAEATMVQPNYTGFLTEPGIYSDAQIE
EWRKIVDAVHKKGGLIFLQLIHAGRAGIPEKILQQPKSDQDPLAGRLLAASAIPIKDHRIPAYFAASGEKETYGVPEELTDDEV
RNGIIPLFVEGAKNAIFKAGFDGVEIHGANGYLLDAFFRESSNKRQSGPYAGTTIDTRCQLIYDVTKSVCDAVGSDRVGLRISP
LNGVHGMIDSNPEALTKHLCKKIEPLSLAYLHYLRGDMVNQQIGDVVAWVRGSYSGVKISNLRYDFEEADQQIREGKVDAV
AFGAKFIANPDLVERAQHDWPLNEPRPETYYTRTAVGYNDYPTYNN

C) NCBI-CD search domain “OYE_like_FMN” from position 6 to 360, E-value 2,8e-163.

MATFPELLRPLKLGRYTLRNRIIMAPLTRCQATEDGHVPRTESMLKYYEDRASAGLIIAEATMVQPNYTGFLTEPGIYSDAQIE
EWRKIVDAVHKKGGLIFLQLIHAGRAGIPEKILQQPKSDQDPLAGRLLAASAIPIKDHRIPAYFAASGEKETYGVPEELTDDEV
RNGIIPLFVEGAKNAIFKAGFDGVEIHGANGYLLDAFFRESSNKRQSGPYAGTTIDTRCQLIYDVTKSVCDAVGSDRVGLRISP
LNGVHGMIDSNPEALTKHLCKKIEPLSLAYLHYLRGDMVNQQIGDVVAWVRGSYSGVKISNLRYDFEEADQQIREGKVDAV
AFGAKFIANPDLVERAQHDWPLNEPRPETYYTRTAVGYNDYPTYNN
